# The Secondary Resistome of Methicillin-Resistant *Staphylococcus aureus* to β-Lactam Antibiotics

**DOI:** 10.3390/antibiotics14020112

**Published:** 2025-01-21

**Authors:** Nader Abdelmalek, Sally Waheed Yousief, Martin Saxtorph Bojer, Mosaed Saleh A. Alobaidallah, John Elmerdahl Olsen, Bianca Paglietti

**Affiliations:** 1 Department of Biomedical Sciences, University of Sassari, 07100 Sassari, Italy; n.abdelmalek@studenti.uniss.it (N.A.); s.yousief@studenti.uniss.it (S.W.Y.); 2 Department of Veterinary and Animal Sciences, Faculty of Health and Medical Sciences, University of Copenhagen, 1870 Frederiksberg C, Denmark; masab@sund.ku.dk (M.S.B.); obaidallahm@ksau-hs.edu.sa (M.S.A.A.); jeo@sund.ku.dk (J.E.O.); 3 Department of Clinical Laboratory Sciences, College of Applied Medical Sciences, King Saud Bin Abdulaziz University for Health Sciences, Jeddah 21423, Saudi Arabia; 4 King Abdullah International Medical Research Center, Jeddah 22384, Saudi Arabia

**Keywords:** *Staphylococcus aureus*, β-lactam resistance, secondary resistome, TraDIS

## Abstract

**Background**: Therapeutic strategies for methicillin-resistant *Staphylococcus aureus* (MRSA) are increasingly limited due to the ability of the pathogen to evade conventional treatments such as vancomycin and daptomycin. This challenge has shifted the focus towards novel strategies, including the resensitization of β-lactams, which are still used as first-line treatments for methicillin-susceptible *Staphylococcus aureus* (MSSA). To achieve this, it is essential to identify the secondary resistome associated with the clinically relevant β-lactam antibiotics. **Methods**: Transposon-Directed Insertion Site Sequencing (TraDIS) was employed to assess conditional essentiality by analyzing the depletion of mutants from a highly saturated transposon library of MRSA USA300 JE2 exposed to ½ minimal inhibitory concentration (MIC) of oxacillin or cefazolin. **Results**: TraDIS analysis led to the identification of 52 shared fitness genes involved in β-lactam resistance that are primarily linked to cell wall metabolism and regulatory systems. Among these, both known resistance factors and novel conditionally essential genes were highlighted. As proof of concept, transposon mutants corresponding to nine genes (*sagB*, *SAUSA300_0657*, *SAUSA300_0957*, *SAUSA300_1683*, *SAUSA300_1964*, *SAUSA300_1966*, *SAUSA300_1967*, *SAUSA300_1692*, and *mazF*) were grown in the presence of β-lactam antibiotics and their MICs were determined. All mutants showed significantly reduced resistance to β-lactam antibiotics. **Conclusions**: This comprehensive genome-wide investigation provides novel insights into the resistance mechanisms of β-lactam antibiotics, and suggests potential therapeutic targets for combination therapies with helper drugs.

## 1. Introduction

Antimicrobial resistance (AMR) poses a significant global health challenge, directly causing 1.14 million deaths in 2021, surpassing the combined death toll of HIV and Parkinson’s disease [1,2,3]. In the same year, methicillin-resistant *Staphylococcus aureus* (MRSA) was identified as a leading global threat, being the only multidrug-resistant pathogen responsible for over 100,000 deaths annually [1]. This threat is further amplified by the emergence of resistance to last-resort therapeutic strategies, such as vancomycin, highlighting the urgent need for the development of new antimicrobials to address this issue [4]. However, substantial costs, insufficient financial incentives, prolonged development timelines, and low success rates in bringing new antibacterial drugs to the market have greatly discouraged major pharmaceutical companies from investing in this field [5].

Considering these challenges, an alternative strategy involves revitalizing the effectiveness of existing antibiotics against multidrug-resistant bacteria [6]. This strategy has gained significant attention since the discovery of clavulanic acid, which has been successfully used to enhance the efficacy of amoxicillin [7]. Building on this concept, a growing number of studies have explored the molecular determinants and products that re-sensitize MRSA to the current therapeutic pipeline [8,9].

Resistance of MRSA to β-lactam antibiotics is mainly mediated by the acquisition of *mecA*, which encodes the penicillin-binding protein (PBP) 2a. This penicillin-insensitive PBP has a unique active site configuration compared to other PBPs, resulting in a reduced ability to bind β-lactam antibiotics [10]. However, β-lactam antibiotics remain the most widely used class of antibacterial agents in the armamentarium of infectious diseases and are recommended for the management of methicillin-sensitive *Staphylococcus aureus* (MSSA) infections [11]. This makes them ideal candidates for a resensitization approach and the use of adjuvants, based on thorough investigations of the molecular basis of resistance to β-lactams beyond the *mecA* gene.

Transposon Directed Insertion Site Sequencing (TraDIS) integrates genome-wide transposon mutagenesis with sequencing technologies to connect genetic information to phenotypic outcomes [12]. This high-throughput method has been applied to various bacterial pathogens to assess gene essentiality and evaluate the relative contribution of each gene to fitness under specific stress conditions, such as antimicrobial treatment [12,13].

In this study, we used TraDIS to identify conditionally essential or fitness genes in the presence of two clinically relevant β-lactams, oxacillin and cefazolin. Using this approach, we were able to confirm previously known genes linked to β-lactam resistance and identify novel fitness genes and pathways that contribute to it. Single-transposon mutants were then used to validate these genes, which resulted in a reduction in resistance or even resensitization of MRSA to both oxacillin and cefazolin.

## 2. Results

### 2.1. TraDIS Metrics

The characteristics of the transposon library of *S. aureus* USA300 JE2 were described in detail in our previous study [14]. For TraDIS analysis, we calculated the number of mapped reads, unique insertion sites (UISs), and the average distance between unique transposon insertions for each replicate under different conditions: the input library (initial transposon mutant pool before any treatment) [14], treatment conditions with oxacillin and cefazolin (Brain Heart Infusion, BHI broth supplemented with the respective β-lactam antibiotic), and the control condition (BHI without antibiotics). An average of 5.5 million reads was used as the cut-off for each replicate, which was equivalent to twice the genome length. After the first round of sequencing and observation of enrichment of certain mutants, we decided to set a higher cut-off for β-lactam replicates [15]. This adjustment was made to mitigate the potential impact of enriched mutants on the accuracy of the analysis, ensuring that the other mutants were not falsely identified as being involved in resistance. Specifically, we aimed to obtain 14 million reads under cefazolin and oxacillin exposure conditions. The TraDIS metrics are presented in Table 1.

### 2.2. The Secondary Resistome to Oxacillin and Cefazolin

Using TraDIS analysis, we identified 52 shared fitness genes (logFC < −2 and q-value < 0.01) following exposure to ½ the minimal inhibitory concentration (MIC) of cefazolin or oxacillin (Table 2). These shared genes were primarily involved in cell wall metabolism (*fmtA*, *mecA*, *pbp4*, *prsA*, *isaA*, *lytH*, *auxA*, and *auxB*) and regulatory systems (*vraR*, *vraS*, *vraT*, *arlR*, *arlS*, *agrA*, *pknB*, and *rsbU*). We also observed the depletion of certain mutants associated with phage-related processes (*SAUSA300_1964*, *SAUSA300_1966*, *SAUSA300_1967*, and *SAUSA300_1969*) (Table 2, Figure S1). Additionally, we identified 125 genes only in the cefazolin dataset and 42 genes in the oxacillin dataset (Table S1A,B). Further examination revealed that a significant number of these dataset genes exhibited depletion, but did not meet the selection thresholds, rendering many statistically insignificant according to our criteria (Table S1C,D).

To enhance our analysis, we conducted a detailed approach, moving beyond the statistical analysis employed by the *tradis_comparison.R* script, to assess genes at both gene and domain-coding levels. This involved examining individual genes to identify specific transposon insertion sites showing either depletion or enrichment, rather than aggregating transposon reads across the entirety of genes. This refined analysis revealed several genes with enrichment in one or a few transposon mutant insertions, accompanied by the depletion of the majority of transposon insertions within the gene.

**Figure 1:**
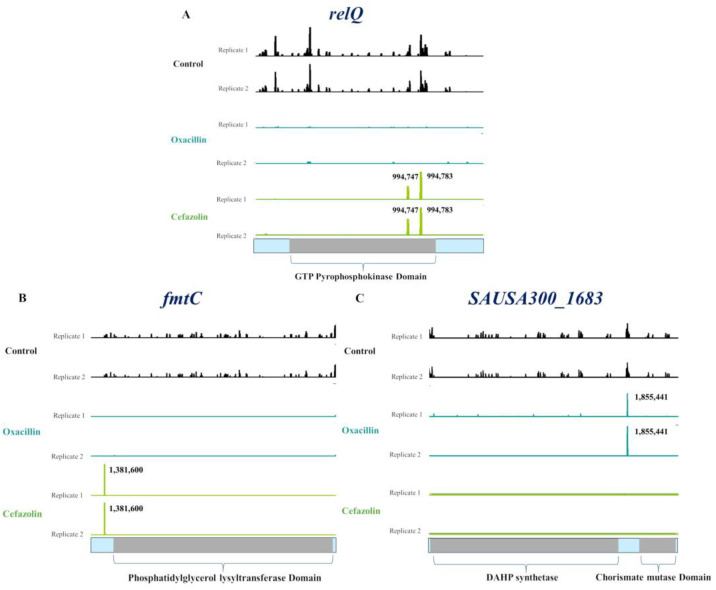
Gene-scale transposon insertion measurements (**A**–**C**). The transposon insertion map of *relQ*, *fmtC*, and *SAUSA300_1683* showed a general depletion of transposon insertions in the presence of oxacillin or cefazolin, compared to the no-antibiotic control. However, there was a notable enrichment of single-transposon insertions at a specific site within *relQ* and *fmtC* in the presence of cefazolin and within *SAUSA300_1683* in the presence of oxacillin. The peaks (control: black; oxacillin: blue; cefazolin: green) represent the frequency of transposon insertions at a given position within the gene.

Therefore, we focused on genes exhibiting a depletion of transposon reads (logFC < −2) in one dataset and an enrichment (logFC > 2) in the other. For instance, we observed a significant depletion in transposon reads of *relQ* and *fmtC* when subjected to oxacillin, contrasting with an unexpected enrichment upon cefazolin exposure with logFC values of 2.3 and 6.9, respectively. This enrichment was particularly evident at two insertion sites within *relQ* (994,747 and 994,783 bp), and at a specific insertion site within *fmtC* (1,381,600 bp) (Figure 1A,B). Conversely, *SAUSA300_1683* transposon reads were depleted during cefazolin exposure, but showed enrichment (logFC: 7.18) in the oxacillin dataset. This enrichment was observed at a single transposon location (1,855,441 bp) (Figure 1C). A similar pattern was also observed for *mazF* (Figure S2).

### 2.3. Validation of Fitness Genes That Reduce Resistance to Oxacillin and Cefazolin

For the validation experiments, 13 genes in which transposon reads were adversely affected by the exposure to oxacillin or cefazolin were selected. Ten of these mutants were chosen based on their statistical significance in both subsets, novelty, and availability of their respective mutants in the Nebraska transposon mutant library [16]. We also included two mutants, *mazF*::Tn and *SAUSA300_1683*::Tn, whose genes were identified using the more detailed gene-/domain-scale approach described above. Additionally, we included the *opp2F*::Tn mutant, where the gene showed a depletion in transposon reads with logFC values of −1.5 and −2.89 in the oxacillin and cefazolin secondary resistome datasets, respectively (Table 2).

**Figure 2:**
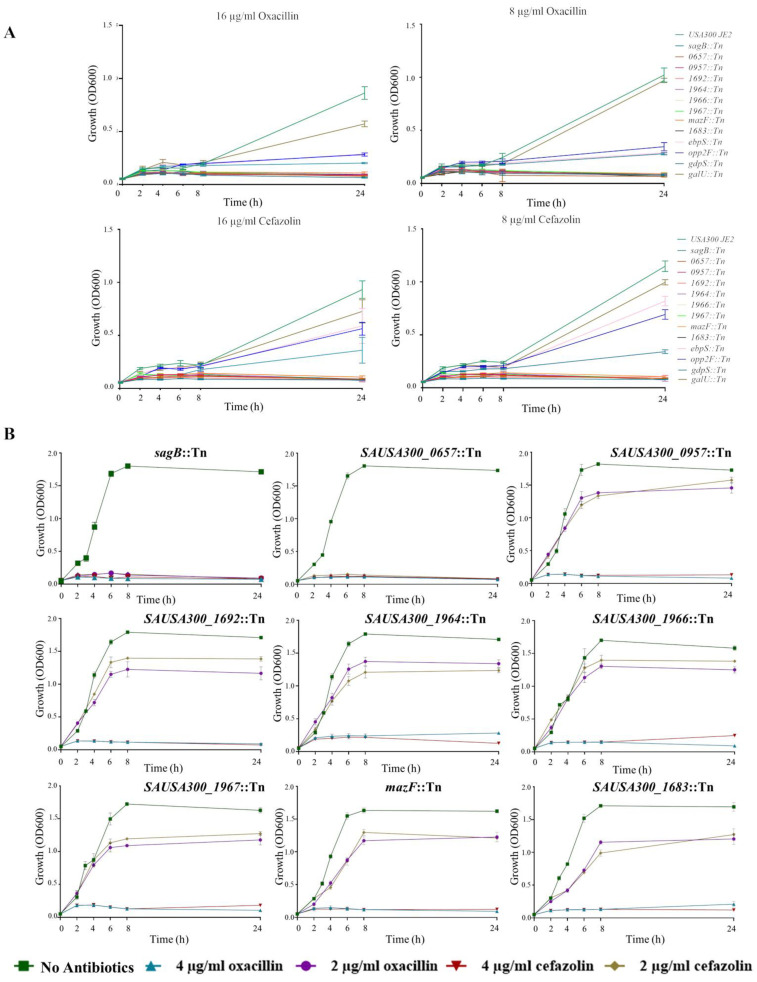
Growth kinetics of WT and selected mutants in the presence of oxacillin or cefazolin. (**A**,**B**) OD600 values were measured in Tryptone Soya broth with or without varying concentrations of oxacillin or cefazolin at six time points (0 h, 2 h, 4 h, 6 h, 8 h, and 24 h). Each data point is based on three biological replicates, with standard deviations represented as error bars.

Transposon mutants were first grown in the absence of antimicrobials. All mutants grew equally well as the wild-type (WT) under this condition (Figure S3). They were then grown in the presence of oxacillin or cefazolin at concentrations of 16 and 8 µg/mL, corresponding to ½ and ^1^/_4_ of the MIC of the WT strain, respectively (Figure 2A). Among the 13 mutants tested, 4 mutants showed either less severe growth defects (*opp2F*::Tn, *gdpS*::Tn and *ebpS*::Tn) or any significant differences (*galU*::Tn) compared to the WT, and were therefore excluded from subsequent analysis (Figure 2A). In contrast, nine mutants (*SAUSA300_0657*::Tn, *sagB*::Tn, *SAUSA300_1964*::Tn, *SAUSA300_1966*::Tn, *SAUSA300_1967*::Tn, *mazF*::Tn, *SAUSA300_957*::Tn, *SAUSA300_1692*::Tn, and *SAUSA300_1683*::Tn) exhibited significant growth defects under these conditions and were then exposed to reduced concentrations of 4 µg/mL (^1^/_8_ MIC) and 2 µg/mL (^1^/_16_ MIC) of oxacillin or cefazolin (Figure 2B). Notably, *sagB*::Tn and *SAUSA300_0657*::Tn mutants displayed growth defects at a lower concentration (2 µg/mL) of both cefazolin and oxacillin (Figure 2B).

To further assess the bactericidal effect, time-kill assays were performed in the presence of 4 µg/mL oxacillin or cefazolin. These assays revealed a significant reduction in colony-forming units (CFUs) in the mutants compared with the WT (Figure S4).

We measured the MICs of the mutant strains using broth microdilutions to evaluate whether growth attenuation led to reduced resistance to oxacillin and cefazolin (Table 3). The inactivation of *sagB* and *SAUSA300_0657* resulted in a 16-fold reduction in the MIC for both tested β-lactams compared to the WT. Additionally, transposon mutants of *SAUSA300_0957*, *mazF*, *SAUSA300_1964*, *SAUSA300_1966*, *SAUSA300_1967*, *SAUSA300_1692*, and *SAUSA300_1683* exhibited increased susceptibility to both oxacillin and cefazolin, with MIC reductions of 4-fold or 8-fold compared to the WT. In contrast, transposon mutants *gdpS*, *ebpS*, *opp2F*, and *galU* showed MICs similar to those of the WT, reflecting their less severe growth defects in the growth assays. In order to understand why these mutants had shown to be depleting during the initial screening, they were subjected to a competition assay in the presence of ½ and ¼ MIC of oxacillin. In this assay, we observed a significant decrease in the mutant/WT ratio for all mutants, except for the *galU* mutant when exposed to ¼ MIC of oxacillin, compared to no antibiotic exposure (Figure S5).

### 2.4. Conservation of Fitness Genes and Homology of Encoded Proteins to the Human Proteome

To identify potential targets for helper drugs, it is essential that the selected genes are conserved across *S. aureus* strains to ensure broad-spectrum applicability and exhibit no homology to the human proteome, thereby minimizing potential off-target effects. Our analysis revealed that six genes (*sagB*, *SAUSA300_0657*, *SAUSA300_0957*, *mazF*, *SAUSA300_1692*, *and SAUSA300_1683*) were highly conserved across all 146 MRSA strains analyzed (Figure S6). In contrast, the phage-related genes (*SAUSA300_1964*, *SAUSA300_1966*, and *SAUSA300_1967*) were not conserved, with conservation percentages ranging from 32% to 76%. Additionally, the proteins encoded by the selected genes showed no homology with the human proteome.

## 3. Discussion

Therapeutic strategies for MRSA have become increasingly limited owing to the ability of the pathogen to evade conventional treatments such as vancomycin and daptomycin [17]. This growing challenge has shifted the focus towards novel strategies, such as antibiotic resensitization to β-lactams [8,9,18]. These antimicrobials are particularly promising agents for this strategy due to their low toxicity and established use as first-line treatments for MSSA infections [19]. Achieving this goal requires a comprehensive understanding of the molecular mechanisms driving β-lactam resistance in MRSA, which has been studied using various investigative approaches [20,21,22]. Resistance to β-lactams is primarily mediated by the *mecA* gene, which encodes transpeptidase PBP2A. This enzyme catalyzes cell wall cross-linking, even in the presence of β-lactam antibiotics, leading to low to intermediate levels of resistance [21]. To achieve high-level resistance, additional determinants are necessary, representing the submerged portion of the iceberg beyond *mecA*, known as the ‘secondary resistome’ [13,21,22]. The term ‘secondary resistome’ refers to a collection of non-essential chromosomal or plasmid-borne genes, known as fitness or auxiliary genes, that become essential for bacterial survival when exposed to sub-inhibitory concentrations of antimicrobials [13].

In this study, we comprehensively investigated the secondary resistome of community-associated MRSA USA300 JE2 using TraDIS, when exposed to ½ MIC of both oxacillin and cefazolin, β-lactams commonly used in clinical practice. This concentration was chosen to ensure the genome-wide nature of our investigation, supported by previous studies demonstrating that low sub-inhibitory concentrations provide limited insight into the resistance determinants involved upon exposure to antibiotics [23,24].

This high-throughput genome-wide approach enabled us to identify 52 shared genes as fitness genes between the oxacillin and cefazolin datasets. Our analysis revealed several genes involved in MRSA resistance to β-lactams, along with *mecA*, including both newly identified and previously known genes, thereby validating our transposon screening approach. The previously identified genes confirmed here are mainly involved in cell wall metabolism and division, such as *fmtA*, *pbp4*, *auxA*, *auxB*, *prsA*, *isaA*, and *lytH*, as well as signal transduction and transcription elements such as the *vraRS* and *arlRS* two-component regulatory systems, *sarA*, *agrA*, *pknB*, and *sigB* [25,26,27,28,29,30]. Previous genome-wide studies have also investigated the fitness genes in the presence of β-lactam antibiotics. For instance, one study manually screened the Nebraska single-transposon mutant library and identified 13 genes involved in oxacillin resistance [31]. Another study explored the MSSA HG001 strain and identified 19 genes as intrinsic resistance factors for oxacillin using transposon sequencing (tn-seq) [32]. Although we observed an overlap with our findings for some fitness genes, such as *auxA*, *auxB*, *sigB*, and *pbp4*, the differences in experimental design and screening methods were substantial. Our approach addresses the limitations of previous studies, such as low-throughput screening and the use of MSSA strains, by accurately identifying and validating several known and novel fitness genes involved in β-lactam resistance that were not identified in the aforementioned studies [31,32].

Building upon previous studies, our research significantly contributes to the existing understanding of MRSA resistance mechanisms by uncovering several newly identified fitness genes that play key roles during β-lactam exposure. Among the newly identified genes that reduce β-lactam resistance upon inactivation, we highlight the depletion of transposon insertions to varying extents in various phage-related genes. These genes are located within the integrated φSa3 prophage, which comprises 55 genes, including a highly human-specific immune evasion cluster [33]. This cluster is predominantly linked to human host pathogenicity and plays an important role in host-switching events in livestock origin strains, such as MRSA ST398 [34]. Although the identified fitness genes in the φSa3 prophage have not been directly linked to β-lactam resistance, a previous transcriptomic study demonstrated the induction of these phage-related genes in the presence of oxacillin [25]. Furthermore, the same study revealed that these genes were downregulated in the *vraS* and *vraT* mutants, which were previously identified and validated in our study as auxiliary factors for β-lactam resistance [25]. Similarly, these phage-related genes were identified as part of the resistome, along with *vraR* and *vraT*, against the antimicrobial peptide LL-37, which interacts with bacterial lipopolysaccharides and lipoteichoic acids, thereby disrupting membrane integrity [35]. Additionally, previous studies have reported that these φSa3 prophage genes are upregulated and might provide an adaptive advantage under vancomycin stress [36]. Transposon inactivation of three φSa3 prophage genes (*SAUSA300_1964*, *SAUSA300_1966*, *and SAUSA300_1967*) resulted in a 4- to 8-fold reduction in the MIC of both oxacillin and cefazolin compared to WT. Therefore, the involvement of these φSa3-harbored genes in β-lactam resistance may be mediated through a *vra*-dependent mechanism, suggesting an interplay between phage-related proteins and the VraRST three-component signaling pathway.

*SAUSA300_0957*, previously identified as essential for survival under salt stress in *S. aureus*, has also been reported to exhibit a slight increase in oxacillin resistance when knocked out in a USA300 LAC background [37]. It is worth noting that the previous study used E-strips in their experimental setup. Conversely, we observed a significant depletion of transposon reads in *SAUSA300_0957* in the presence of ½ MIC of oxacillin and cefazolin. This finding was validated as the transposon mutant *SAUSA300_0957*::Tn exhibited defective growth in the presence of β-lactams and a 4-fold decrease in MIC for both oxacillin and cefazolin. Notably, *SAUSA300_0957* is located upstream of *fmtA*, a gene known to be involved in β-lactam resistance [38]. Further investigations are necessary to explore the potential link between *SAUSA300_0957* and the adjacent genes involved in cell wall metabolism in response to β-lactams.

Toxin–antitoxin (TA) systems in *S. aureus* play important roles in bacterial survival and pathogenicity [39]. The MazEF module is among the well-studied TA systems. MazE neutralizes the toxic effects of MazF, an mRNA interferase that cleaves mRNA at specific sequences. This cleavage leads to a reduction in protein synthesis and consequently inhibits bacterial growth [39]. Deletion of *mazEF* in an MSSA strain has been linked to increased sensitivity to β-lactam antibiotics [40]. Furthermore, a previous study showed that inactivation of *mazE* resulted in the upregulation of *mecA* and an increased MIC for oxacillin in a clinical MRSA isolate [41]. Consistent with these findings, we observed a depletion of transposon reads in *mazF*, which was validated by an 8-fold reduction in the MIC of the *mazF*::Tn mutant for both oxacillin and cefazolin.

An additional key factor in β-lactam resistance is the stringent response, as highlighted in several studies [42,43]. This response significantly contributes to the resistance phenotype by transforming an initially heterogeneous population, in which only a small fraction of bacterial cells exhibit high resistance to β-lactams, into a homogeneous and highly resistant population [43]. The guanine-derived second messenger (p)ppGpp modulates the stringent response in *S. aureus* and other bacteria, enabling them to adapt to various environmental challenges, including β-lactam stress [43]. Exposure to β-lactams triggers increased synthesis of (p)ppGpp from guanosine triphosphate (GTP) by GTP pyrophosphokinases, leading to reduced GTP levels [44]. This decrease in intracellular GTP levels de-represses CodY, thereby inducing a stringent response [44].

Interestingly, *SAUSA300_0657* encodes a hypothetical protein containing a DUF402 domain. A previous study structurally and functionally characterized the DUF402 domain of *SAUSA300_1848*, revealing its nucleoside tri- and diphosphate hydrolysis activity, with a preference for GTP [45]. Additionally, it has been reported that the catalytic hydrolysis center in this domain is conserved among DUF402 proteins [43]. The *SAUSA300_0657*::Tn mutant showed a 16-fold decrease in the MICs of β-lactams within the susceptibility range (MIC ≤ 2 µg/mL), confirming the TraDIS results. Based on our observations and Wang et al.’s hypothesis, we propose that the inactivation of genes coding for the DUF402 domain reduces β-lactam resistance, likely by preventing the induction of the stringent response as explained above [45]. Unfortunately, we could not assess the involvement of *SAUSA300_1848* because of its low representation in the control replicates, with no transposon mutant available in the Nebraska transposon library. Furthermore, *SAUSA300_0657* has been previously identified as an auxiliary factor for resistance to both dalbavancin and daptomycin [24,46]. These antibiotics target the bacterial cell wall, and the determinants of daptomycin resistance also involve the stringent response [46,47]. Another gene identified here as a fitness gene, *SAUSA300_1692*, has been previously reported to play a role in guanine transport [48]. Supplementation with guanine or guanosine has been shown to reduce oxacillin resistance in MRSA by increasing the flux into the GTP pathway in a dose-dependent manner [49]. Taken together, these findings suggest that genes involved in GTP homeostasis are potential candidates for the development of adjuvant therapies to enhance the efficacy of β-lactams.

*sagB* encodes a membrane-associated N-acetylglucosaminidase that cleaves the polymerized glycan chains of peptidoglycans to their physiological length [50]. Deletion of *sagB* in *S. aureus* has been shown to cause morphological defects owing to the excessive length of peptidoglycan strands [50]. In addition to its well-understood function, *sagB* has been reported as a part of the secondary resistome to oxacillin in MRSA [51]. Interestingly, the same study noted an enrichment of transposon reads upon cefoxitin stress, suggesting that the inactivation of this gene may increase cefoxitin tolerance and potentially resistance [51]. These observations, along with our results, prompted us to investigate whether the efficacy of cefazolin can be restored by *sagB* inactivation. We observed the expected pattern, with a 16-fold reduction in the MIC of oxacillin and cefazolin. Furthermore, oxacillin primarily targets PBP2 and PBP3, cefazolin targets PBP1 and PBP2, while cefoxitin specifically targets PBP4 [51,52]. This suggests that *sagB* may act as an auxiliary factor for β-lactam antibiotics in a PBP-dependent manner.

While the BioTradis tool is valuable for analyzing raw TraDIS data to identify essential and fitness genes, our research demonstrates that genes such as *mazF* and other known genes involved in β-lactam resistance, like *fmtC* and *relQ*, were not identified in at least one of the treatment conditions [53,54]. Using a more detailed gene-/domain-scale analysis, we observed an enrichment of specific transposon insertions alongside the depletion of the majority of transposon reads in these genes. Taking *relQ* as an example, we observed a significant depletion (LogFC: −2.6) during oxacillin exposure, in contrast to the enrichment (LogFC: 2.3) of two transposon insertion site reads (994,747 and 994,783) in the presence of ½ MIC of cefazolin. These insertions are located at the position encoding the second-to-last alpha-helix at the C-terminus, which forms the homotetramer interface of RelQ [55]. These two amino acids are structurally critical, as RelQ has always been described as a strict tetramer and cannot exist as a monomer [55]. We hypothesize that the enrichment of *relQ* mutants with a single-transposon insertion might be due to a secondary mutation that compensates for the loss of (p)ppGpp synthesis by RelQ. Therefore, we recommend adopting a more detailed approach such as our gene/domain approach to better detect and analyze patterns overlooked by the statistical methodology employed by the Biotradis tool. This approach aims to mitigate the risk of overlooking or inaccurately classifying fitness genes, a common issue stemming from the reliance on statistical criteria that may lead to the exclusion of biologically significant genes due to cut-off values [56].

In addition to the point mentioned above, the limitations of TraDIS, such as not taking into consideration the role of essential genes and the competitive nature of screening experiments, have been previously described [23,57]. These limitations are evident in our current work, as we identified false-positive fitness genes, such as *galU*. Although screening the transposon mutant library at both low and varied concentrations could address these issues by improving the statistical significance of the data, it may also reduce the number of identified resistance determinants and fail to uncover new fitness genes [23,24].

In summary, this study provided a comprehensive genome-wide investigation of the secondary resistome to both oxacillin and cefazolin, highlighting the multifaceted mechanisms involved. Our findings demonstrate that inactivating secondary resistance genes can reduce resistance and even re-sensitize MRSA to β-lactam antibiotics. Importantly, we confirmed and expanded the known secondary resistome, and identified novel genes that could potentially be targeted. These insights pave the way for future research to develop or test existing helper drugs, that could reintroduce these antibiotics into the therapeutic arsenal against MRSA.

## 4. Materials and Methods

### 4.1. Bacterial Strains and Culture Conditions

The strains used in the present study are listed in Table 4. Both WT and mutant strains of *S. aureus* were grown in Brain Heart Infusion (BHI) broth or on BHI agar, Tryptone Soya broth (TSB), Tryptone Soya agar, or cation-adjusted Mueller Hinton broth (CAMHB) (Microbiol, Cagliari, Italy) supplemented with 2% NaCl (Sigma-Aldrich, St. Louis, MO, USA). Oxacillin, cefazolin, and erythromycin (Sigma-Aldrich) were appropriately added to the medium.

### 4.2. TraDIS Library Antimicrobial Exposure and DNA Extraction

An aliquot of a high-density transposon mutant library of USA300 JE2 [14], containing 2.7 × 10^9^ CFU/mL, was inoculated in 10 mL of BHI with or without ½ minimum inhibitory concentration of oxacillin and cefazolin, separately, at 37 °C until an OD600 of 0.5 was reached. Both control and screening experiments were performed in duplicate to ensure biological replicates for further bioinformatic analysis. Genomic DNA was extracted using the GenElute bacterial genomic DNA kit (Sigma-Aldrich, St. Louis, MO, USA) according to the manufacturer’s instructions, except for the addition of 200 units/mL of lysostaphin (Sigma-Aldrich) to facilitate staphylococcal cell wall lysis. NanoDrop 1000 (Thermo Fisher Scientific, Waltham, MA, USA) was used to evaluate the quality of the DNA extractions, ensuring their suitability for TraDIS, and the concentration of DNA was determined using the Qubit dsDNA HS assay kit (Thermo Fisher Scientific).

### 4.3. Multiplexed TraDIS

Multiplexed TraDIS for the control and output libraries was performed following the methodology described by Barquist et al. [58]. Briefly, 2 μg of purified DNA was resuspended in 100 μL of molecular-biology-grade water and mechanically sheared using an ultrasonicator (Bioruptor^®^ Pico, Diagenode, Liège, Belgium) to an average size of 300 bp, which was confirmed using the High-Sensitivity DNA Kit in the Agilent 2100 Bioanalyzer System (Agilent Technologies, Santa Clara, CA, USA). Fragmented DNA was converted to blunt-ended DNA using the NEBNext end repair module (New England Biolabs, Ipswich, MA, USA) and dA-tailed using the NEBNext dA-tailing module (New England Biolabs). To enrich transposon-containing fragments more efficiently, a splinkerette approach was employed by ligating SpIA5 adaptors using the NEBNext Quick Ligation module. Following ligation, fragments containing the transposon sequence were enriched by PCR using transposon-specific and adapter primers (SplAP5.x) (Table S2). After each step of library preparation, DNA fragments were cleaned and size-selected using Agencourt AMPure XP beads (Beckman Coulter, Brea, CA, USA). Prior to sequencing, libraries were quantified and qualitatively verified using SYBR Green qPCR and a Bioanalyzer (Agilent Technologies). DNA libraries were sequenced using a MiSeq 50-cycle V2 reagent kit and flow cells (Illumina, San Diego, CA, USA), according to the TraDIS recipe, as previously described [58,59].

### 4.4. Bioinformatics

Sequence reads from the control and test libraries were analyzed and processed using the BioTraDIS pipeline available online (https://github.com/sanger-pathogens/Bio-Tradis, accessed on 1 February 2024) [58]. The *Bacteria_tradis* script was used to filter and remove tags (TAAGAGACAG) from the resulting reads, which were then mapped to the reference genome (GenBank accession number: CP000255.1) using SMALT 0.7.6. Consequently, unique transposon insertion sites and the number of reads were determined for each gene. The *tradis_comparisons*.R script was used to determine the differences in insertions between the antimicrobial-exposed and unexposed samples. Fitness genes were assigned based on log fold change (logFC) cut-off values of <−2, q-value < 0.01, and logCPM > 1.5. Transposon insertion reads were further measured at the gene scale using the *gene_domain_scale*.R script, which counts the number of reads within each gene. This script can be modified to measure the number of reads in any specified range or focus on the domain-coding region. The results were subsequently visualized using the Artemis genome viewer version 18.2.0. The selected genes were re-annotated using the UniProt database and functionally characterized using the eggNOG-mapper 2.1.9.

### 4.5. Antimicrobial Susceptibility Testing, Growth Experiments, and Time-Kill Assays

Antimicrobial susceptibility testing was performed by broth microdilution in CAMHB + 2% NaCl, following the CLSI standards [60]. MICs were recorded after 24 h of incubation at 37 °C. Growth curves and time-kill assays were performed for each mutant and WT in triplicate in TSB with or without sub-inhibitory concentrations of antibiotics, using a starting inoculum of approximately 10^6^ CFU/mL.

### 4.6. In Silico Conservation and Homology Analysis

Gene conservation analysis was performed on 146 MRSA strains obtained from the National Center for Biotechnology Information (NCBI) gene database. Fitness gene sequences were mapped against these 146 strains using the NCBI Basic Local Alignment Search Tool, BLAST+ (version 2.9.0). The protein sequences of the fitness genes were then compared to those of the human proteome (taxid: 9606) using BLAST+ (version 2.9.0). Proteins that did not yield significant matches within the human proteome at an E-value cutoff of 10 × 10^−10^ were classified as non-homologous [56].

## Figures and Tables

**Table 1:** Summary of TraDIS metrics.

	No. of Reads	Reads Mapped (%) ^1^	UISs ^2^	Total Sequence Length/Total UIS ^3^
Input (Replicate 1) ^4^	14,654,230	78.39	315,400	9.10
Input (Replicate 2) ^4^	10,210,994	79.6	392,797	7.31
Control (Replicate 1)	6,788,911	84.32	466,380	6.15
Control (Replicate 2)	8,167,660	94.14	429,496	6.68
Oxacillin (Replicate 1)	8,621,531	81.20	156,112	18.40
Oxacillin (Replicate 2)	8,508,941	86.55	110,275	26.05
Cefazolin (Replicate 1)	9,412,621	93.78	76,586	37.51
Cefazolin (Replicate 2)	7,351,940	79.71	73,881	38.88

^1^ Number of mapped sequence reads against *S. aureus* USA300 JE2 genome (CP000255.1) (% of the raw data). ^2^ Number of Unique Insertions Sites (UISs). ^3^ The average distance between transposon insertions. ^4^ The initial transposon library (input) was previously obtained and analyzed [14].

**Table 2:** Predicted fitness genes in USA300 JE2 by *tradis_comparison*.R in the presence of ½ MIC of oxacillin and cefazolin.

Locus Tag	Genes	Description	Oxacillin		Cefazolin	
			LogFC	q-value	LogFC	q-value
Cell wall Metabolism						
SAUSA300_0959	*fmtA*	autolysis and methicillin resistance-related protein FmtA	−10.34	1.22 × 10^−13^	−2.21	0.0002
SAUSA300_1720	***sagB***	Peptidoglycan hydrolase	−8.55	1.48 × 10^−07^	−3.43	0.0005
SAUSA300_1588	*lytH*	N-acetylmuramoyl-L-alanine amidase	−4.91	8.8 × 10^−14^	−2.19	7.25 × 10^−05^
SAUSA300_1790	*prsA*	foldase protein PrsA precursor	−4.56	4.59 × 10^−09^	−2.36	3.61 × 10^−07^
SAUSA300_1003	*auxB*	putative membrane protein	−4.01	4.99 × 10^−13^	−2.05	2.67 × 10^−08^
SAUSA300_0032	*mecA*	penicillin-binding protein 2′	−3.53	3.61 × 10^−11^	−3.57	2.23 × 10^−50^
SAUSA300_0980	*auxA*	putative membrane protein	−3.84	2.12 × 10^−11^	−2.92	1.46 × 10^−14^
SAUSA300_1370	***ebpS***	cell surface elastin binding protein	−3.23	7.26 × 10^−09^	−2.99	2.93 × 10^−08^
SAUSA300_2439	***galU***	UTP-glucose−1-phosphate uridylyltransferase	−3.17	1.01 × 10^−07^	−2.26	2.15 × 10^−05^
SAUSA300_2506	*isaA*	immunodominant staphylococcal antigen A precursor	−2.73	3.95 × 10^−07^	−2.24	1.85 × 10^−06^
SAUSA300_0629	*pbp4*	penicillin-binding protein 4	−2.53	1.3 × 10^−09^	−4.05	4.15 × 10^−27^
SAUSA300_2078	*murA*	UDP-N-acetylglucosamine carboxyvinyltransferase	−2.24	3.69 × 10^−05^	−2.73	1.01 × 10^−51^
SAUSA300_2249	*ssaA*	secretory antigen precursor SsaA	−2.96	1.33 × 10^−10^	−2.33	4.83 × 10^−15^
Cellular regulatory metabolism						
SAUSA300_1865	*vraR*	DNA-binding response regulator	−5.47	2.24 × 10^−22^	−3.49	2.29 × 10^−23^
SAUSA300_0730	***gdpS***	GGDEF domain protein	−4.58	7.38 × 10^−12^	−2.11	2.46 × 10^−05^
SAUSA300_1866	*vraS*	two-component sensor histidine kinase	−4.23	4.08 × 10^−15^	−4.03	1.84 × 10^−54^
SAUSA300_1867	*vraT*	conserved hypothetical protein	−4.11	1.33 × 10^−20^	−3.24	5.87 × 10^−28^
SAUSA300_1113	*pknB*	serine/threonine protein kinase	−3.58	4.48 × 10^−11^	−3.87	3.41 × 10^−22^
SAUSA300_0350	*SAUSA300_0350*	transcriptional regulator, Cro/CI family-related protein	−3.62	0.002513	−8.03	0.000153
SAUSA300_1992	*agrA*	accessory gene regulator protein A	−2.89	1.59 × 10^−10^	−2.37	3.42 × 10^−26^
SAUSA300_1307	*arlS*	sensor histidine kinase protein	−2.84	2.05 × 10^−11^	−2.67	2.37 × 10^−19^
SAUSA300_1308	*arlR*	DNA-binding response regulator	−2.82	3.79 × 10^−07^	−3.56	1.83 × 10^−22^
SAUSA300_2022	*rpoF*	RNA polymerase sigma-37 factor	−2.77	9.71 × 10^−11^	−2.14	3.61 × 10^−13^
SAUSA300_1842	*SAUSA300_1842*	transcriptional regulator, Fur family	−2.53	1.56 × 10^−05^	−2.5	1.18 × 10^−07^
SAUSA300_1257	*msrR*	peptide methionine sulfoxide reductase regulator MsrR	−2.07	0.002	−2.97	3.17 × 10^−05^
SAUSA300_2025	*rsbU*	sigma-B regulation protein	−2.4	3.59 × 10^−08^	−2.65	1.51 × 10^−24^
SAUSA300_0475	*SAUSA300_0475*	SpoVG protein	−4.34	4.59 × 10^−12^	−2.48	1.18 × 10^−07^
Translation and post-translational modifications						
SAUSA300_1117	*rpmB*	50S ribosomal protein L28	−2.76	0.001405	−3.6	0.00052
SAUSA300_1668	*SAUSA300_1668*	OsmC/Ohr family protein	−2.11	2.56 × 10^−06^	−2.79	8.1 × 10^−11^
SAUSA300_1688	*SAUSA300_1688*	phenylalanyl-tRNA synthetase (beta subunit)	−2.08	4.78 × 10^−05^	−2.41	2.37 × 10^−09^
SAUSA300_1365	*rpsA*	30S ribosomal protein S1	−2	1.01 × 10^−05^	−2.32	1.06 × 10^−26^
Phage-related genes						
SAUSA300_1967	***SAUSA300_1967***	conserved hypothetical phage protein	−8.43	9.13 × 10^−06^	−8.38	2.77 × 10^−05^
SAUSA300_1964	***SAUSA300_1964***	conserved hypothetical phage protein	−3.61	4.49 × 10^−05^	−9.62	1.85 × 10^−10^
SAUSA300_1969	*SAUSA300_1969*	phi77 ORF011-like protein, phage transcriptional repressor	−2.26	0.00067	−2.36	0.00088
SAUSA300_1966	***SAUSA300_1966***	conserved hypothetical phage protein	−2.09	0.0001	−4.93	1.76 × 10^−10^
Others						
SAUSA300_0281	*esaB*	component of the T7SS	−2.59	6.99 × 10^−09^	−2.04	2.88 × 10^−11^
SAUSA300_1067	*psmβ1*	phenol-soluble modulin	−2.37	0.00025	−3.18	1.21 × 10^−05^
SAUSA300_2026 *	***mazF***	mRNA interferase	0.01	1	−3.9	9.33 × 10^−16^
SAUSA300_1273 *	***opp2F***	oligopeptide permease, ATP-binding protein	−1.5	0.0001	−2.89	1.69 × 10^−18^
Unknown						
SAUSA300_0957	***SAUSA300_0957***	conserved hypothetical protein	−3.93	1.76 × 10^−09^	−2.42	1.58 × 10^−10^
SAUSA300_1746	*SAUSA300_1746*	conserved hypothetical protein	−3.65	0.00406	−8.06	0.000317
SAUSA300_0657	***SAUSA300_0657***	conserved hypothetical protein	−3.33	2.5 × 10^−12^	−2.53	1 × 10^−23^
SAUSA300_2297	*SAUSA300_2297*	conserved hypothetical protein	−3.04	6.9 × 10^−12^	−2.29	7.85 × 10^−10^
SAUSA300_2544	*SAUSA300_2544*	conserved hypothetical protein	−2.6	8.77 × 10^−08^	−2.5	1.11 × 10^−11^
SAUSA300_1187	*SAUSA300_1187*	conserved hypothetical protein	−2.44	5.02 × 10^−08^	−2.02	1.69 × 10^−10^
SAUSA300_0942	*SAUSA300_0942*	conserved hypothetical protein	−2.43	1.64 × 10^−06^	−2.03	1.83 × 10^−07^
SAUSA300_2481	*SAUSA300_2481*	conserved hypothetical protein	−2.19	7.74 × 10^−05^	−4.07	6.22 × 10^−09^
SAUSA300_1692	***SAUSA300_1692***	conserved hypothetical protein	−2.16	8.41 × 10^−05^	−5.35	1.04 × 10^−12^
SAUSA300_2272	*SAUSA300_2272*	conserved hypothetical protein	−2.13	2.61 × 10^−06^	−2.67	1.94 × 10^−11^
SAUSA300_0792	*SAUSA300_0792*	conserved hypothetical protein	−2.09	3.09 × 10^−05^	−2.33	3.89 × 10^−06^
SAUSA300_1366	*SAUSA300_1366*	conserved hypothetical protein	−2.05	0.00126	−4.09	2.62 × 10^−06^
SAUSA300_0768	*SAUSA300_0768*	conserved hypothetical protein	−2.03	0.00253	−2.45	0.00127
SAUSA300_0080	*SAUSA300_0080*	conserved hypothetical protein	−2.03	3.99 × 10^−05^	−3.78	7.74 × 10^−10^
SAUSA300_0578	*SAUSA300_0578*	conserved hypothetical protein	−2.02	0.00032	−2.09	0.00534

LogFC: log2 fold change; q-value: adjusted *p*-value; *: Identified only in one subset. Gene names presented in green (bold) were selected for validation.

**Table 3:** MIC values (µg/mL) of oxacillin and cefazolin in WT and selected transposon mutants.

Strain	MIC (µg/mL) of Oxacillin	MIC (µg/mL) of Cefazolin
USA300 JE2 WT	32	32
*sagB::*Tn	2	2
*SAUSA300_0657::*Tn	2	2
*SAUSA300_0957::*Tn	4	4
*mazF::*Tn	4	4
*SAUSA300_1964::*Tn	4	4
*SAUSA300_1966::*Tn	4	4–8
*SAUSA300_1967::*Tn	4	4
*SAUSA300_1692::*Tn	4	4
*SAUSA300_1683::*Tn	4–8	8
*gdpS::*Tn	32	32
*ebpS::*Tn	32	32
*opp2F::*Tn	32	32
*galU::*Tn	32	32

**Table 4:** Bacterial strains used in this study.

Strain	Description	Reference
USA300 JE2	USA300 CA-MRSA isolate cured of p01 and p03 plasmids/parent strain of the Nebraska Transposon Mutant Library	[16]
NE1909 (*sagB*::Tn)	USA300 JE2 containing a transposon insertion in *sagB*. Erm^r^	[16]
NE1654 (*SAUSA300_0657*::Tn)	USA300 JE2 containing a transposon insertion in *SAUSA300_0657*. Erm^r^	[16]
NE1276 (*SAUSA300_1683*::Tn)	USA300 JE2 containing a transposon insertion in *SAUSA300_1683*. Erm^r^	[16]
NE1384 (*SAUSA300_0957*::Tn)	USA300 JE2 containing a transposon insertion in *SAUSA300_0957*. Erm^r^	[16]
NE1340 (*SAUSA300_1692*::Tn)	USA300 JE2 containing a transposon insertion in *SAUSA300_1692*. Erm^r^	[16]
NE1298 (*SAUSA300_1964*::Tn)	USA300 JE2 containing a transposon insertion in *SAUSA300_1964*. Erm^r^	[16]
NE1786 (*SAUSA300_1966*::Tn)	USA300 JE2 containing a transposon insertion in *SAUSA300_1966*. Erm^r^	[16]
NE1371 (*SAUSA300_1967*::Tn)	USA300 JE2 containing a transposon insertion in *SAUSA300_1967*. Erm^r^	[16]
NE1833 (*mazF*::Tn)	USA300 JE2 containing a transposon insertion in *mazF*. Erm^r^	[16]
NE962 (*gdpS*::Tn)	USA300 JE2 containing a transposon insertion in *gdpS*. Erm^r^	[16]
NE1561 (*ebpS*::Tn)	USA300 JE2 containing a transposon insertion in *ebpS*. Erm^r^	[16]
NE614 (*galU*::Tn)	USA300 JE2 containing a transposon insertion in *galU*. Erm^r^	[16]
NE1609 (*opp2F*::Tn)	USA300 JE2 containing a transposon insertion in *opp2F*. Erm^r^	[16]

## Data Availability

Raw sequencing data and metadata are available in the NCBI Sequence Read Archive (SRA), under BioProject accession no. PRJNA1190087.

## Supplementary Materials

The following supporting information can be downloaded at: https://www.mdpi.com/article/10.3390/antibiotics14020112/s1, Figure S1. Transposon reads distribution in phage-related genes. Figure S2. Transposon reads distribution in *mazF*. Figure S3. Growth curves of WT and transposon mutants in TSB. Figure S4. Time-kill kinetics assays for WT and selected mutants in the presence oxacillin and cefazolin. Figure S5. Competition assay of WT and mutants with and without oxacillin. Figure S6. Gene conservation analysis across MRSA strains. Table S1. A: Predicted fitness-genes identified by *tradis_comparison.R* during oxacillin exposure; B: Predicted fitness-genes identified by *tradis_comparison.R* during cefazolin exposure; C: Analysis of fitness genes identified only within the oxacillin dataset; D: Analysis of fitness genes identified only within the cefazolin dataset. Table S2. List of primers used in this study.
