# Supplementary material for: The Secondary Resistome of Methicillin-Resistant Staphylococcus aureus to β-Lactam Antibiotics
Source: Antibiotics (Basel). 2025 Jan 21;14(2):112. doi: 10.3390/antibiotics14020112 (PMC11851648; doi:10.3390/antibiotics14020112)
Supplement: Supplementary file 1 [file antibiotics-14-00112-s001.zip › Supplementary_material_FigureS1S6_TableS2_rev.pdf]

The Secondary Resistome of methicillin-resistant *Staphylococcus aureus* to β-Lactam Antibiotics

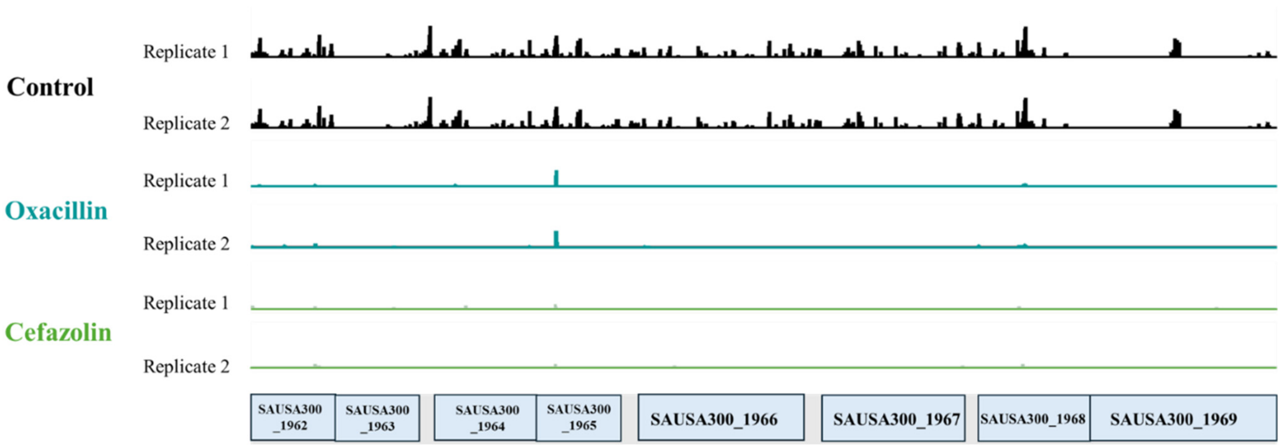

Figure S1. Transposon reads distribution in phage-related genes. This plot, generated using the Artemis genome viewer (18.2.0), illustrates the depletion of transposon insertion in the phage-related region during growth in the presence of oxacillin and cefazolin.

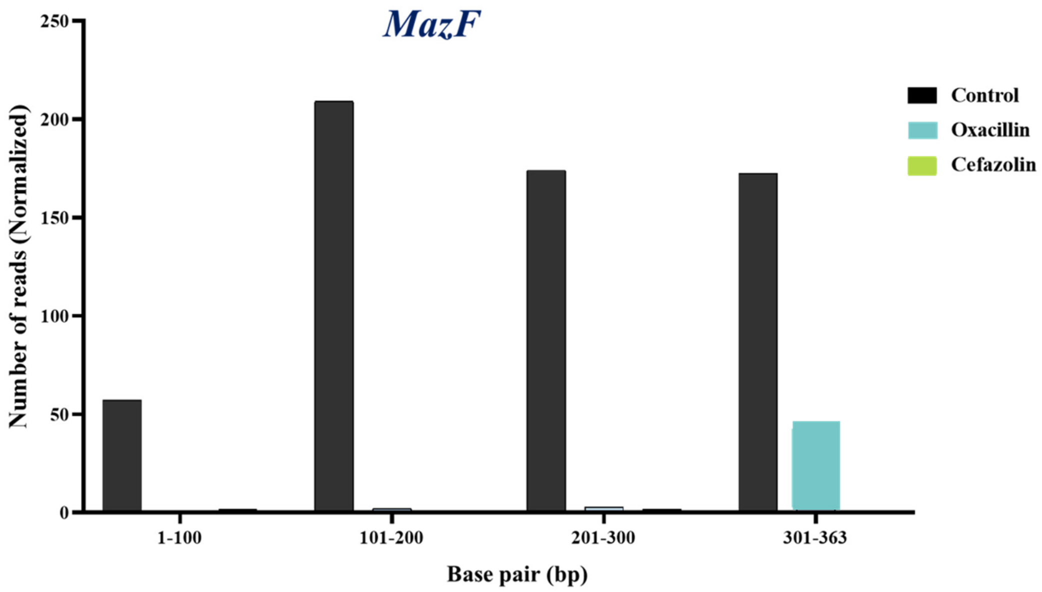

Figure S2. Transposon reads distribution in *mazF*. This bar chart illustrates the number of transposon reads within different regions of the *mazF* gene under three different conditions: control (black bars), oxacillin (blue bars), and cefazolin (green bars). The chart reveals that for one particular insertion in the C-terminal region, there is an elevated number of reads for one replicate during oxacillin exposure compared to other regions or treatments.

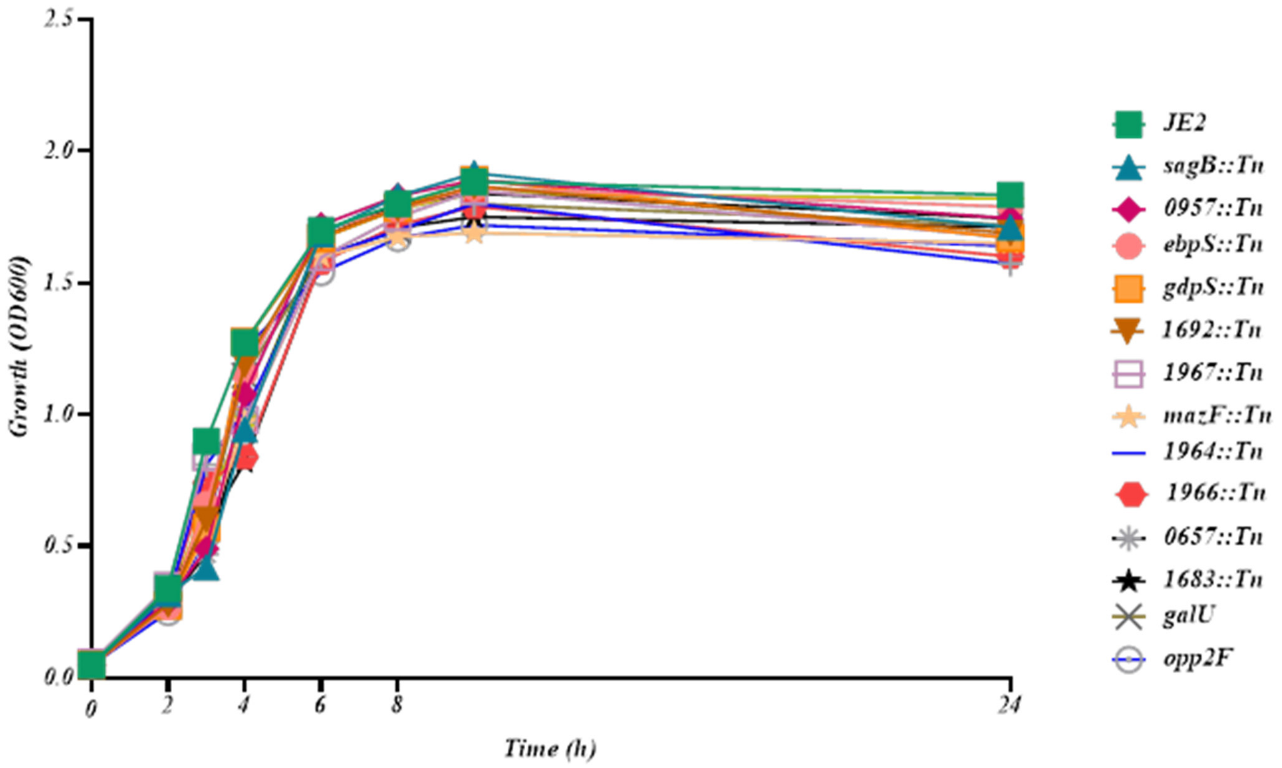

Figure S3. Growth curves of WT and transposon mutants in TSB. Cultures of WT and transposon mutants were incubated in TSB at 37°C. Optical density at 600 nm (OD600) was measured at various time points (0 h, 2 h, 4 h, 6 h, 8 h, and 24 h). Each strain was tested in three biological replicates.

A

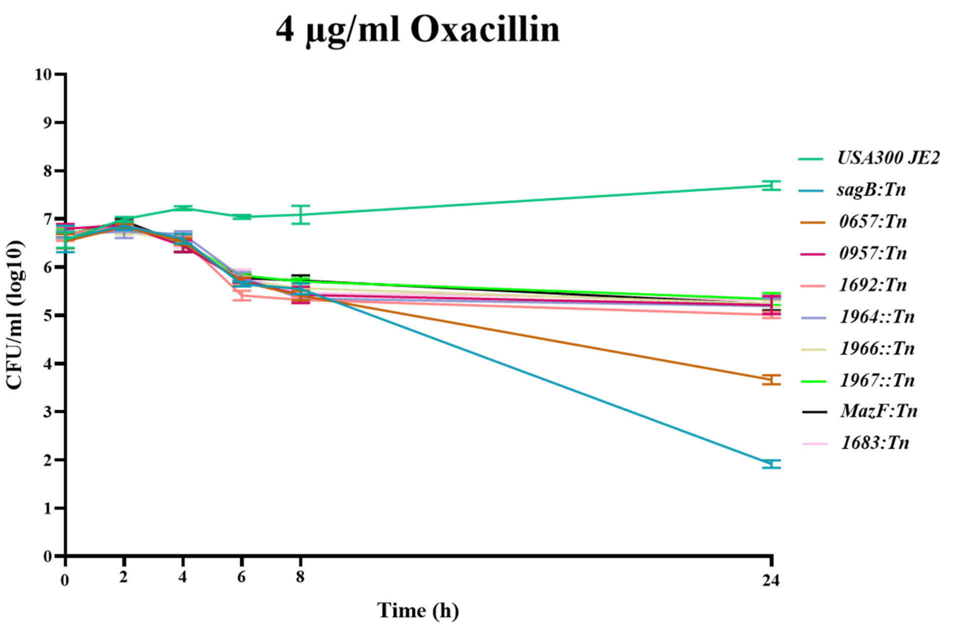

B

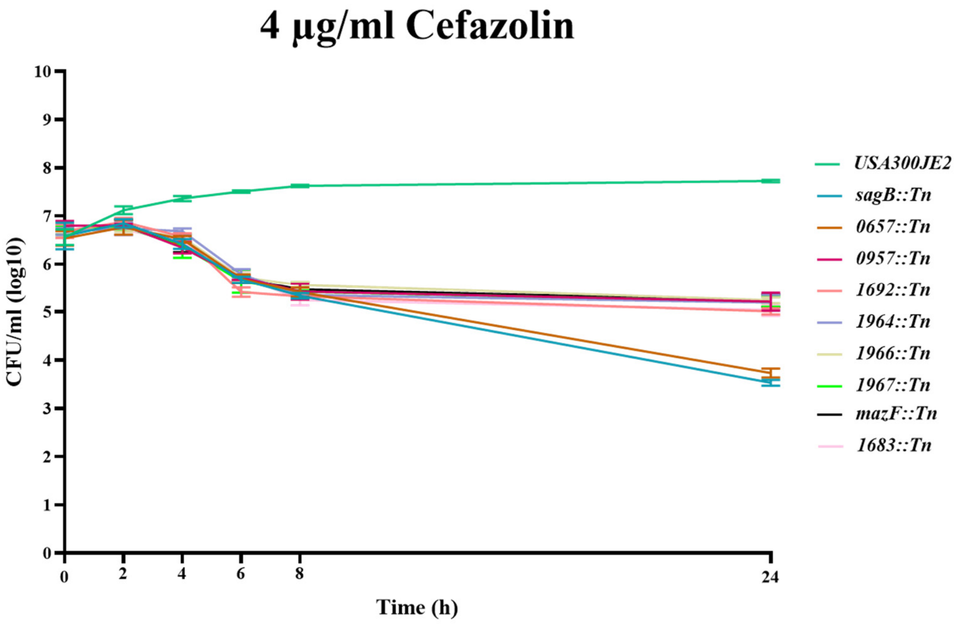

Figure S4. Time-kill kinetics assays for WT and selected mutants in the presence oxacillin and cefazolin. The activity of oxacillin (A) and cefazolin (B) was assessed by measuring the CFU/mL at various time points (0 h, 2 h, 4 h, 6 h, 8 h, and 24 h) in the presence of 4 µg/mL of the antimicrobial. Mean log10 CFU/mL values are displayed, with standard deviations indicated by error bars.

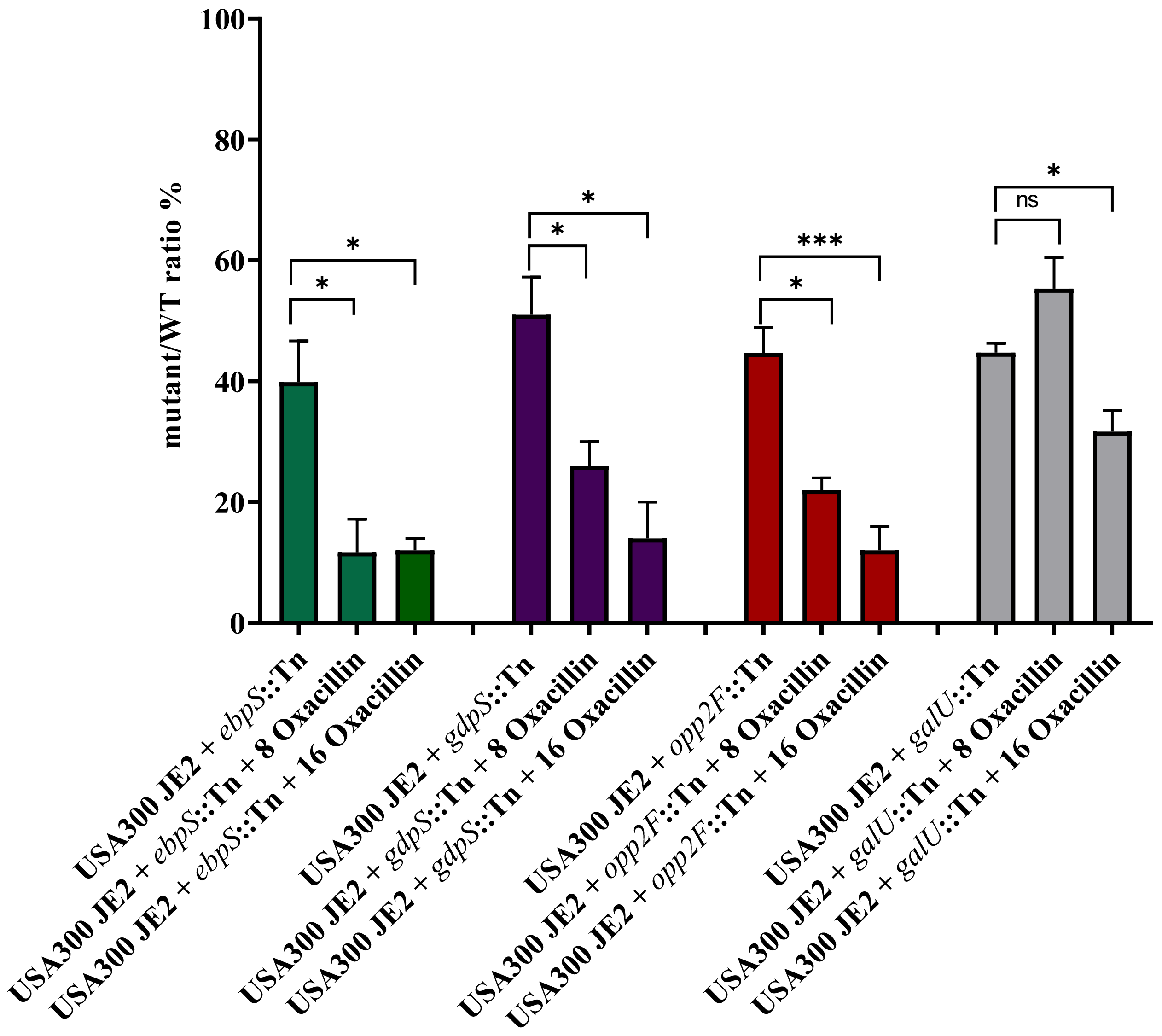

Figure S5. Competition assay of WT and mutants with and without oxacillin. Competition between transposon mutants and the parental strain was assessed in the presence and absence of 8 and 16 µg/ml oxacillin. The proportion of mutants to wild-type (WT) was determined by counting colony-forming units (CFUs) on TSA plates, with and without 5 mg/L erythromycin, following a 24-hour co-culture experiment. Three biological replicates were performed for each condition. The statistical significance was determined using the paired t-test. Asterisks '*' and '***' correspond to an adjusted p-value < 0.05 and p-value <0.001, respectively.

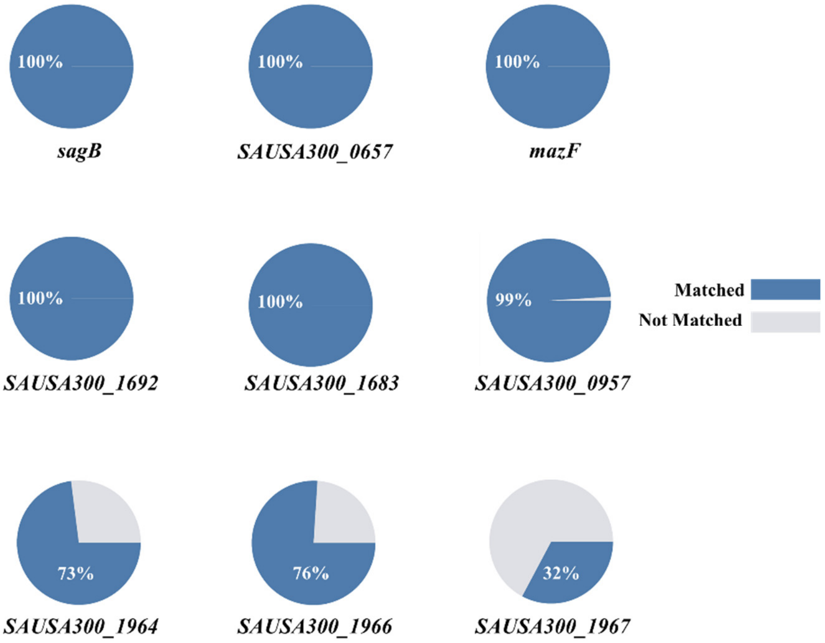

Figure S6. Gene conservation analysis across MRSA strains. These pie charts illustrate the percentage of conservation of fitness genes involved in β-lactam resistance across 146 MRSA strains retrieved from the NCBI database.

Table S2. List of primers used in this study

Primer's Name	Sequence (5'-3')	Reference
SplA5-Top	G*AGATCGGTCTCGGCATTCCTGCTGAACCGCTCTTCC GATC*T	[58]
SplA5-Bottom	/5PHOS/G*ATCGGAAGAGCGGTTCAGCAGGTTTT TTTTTTCAAAAAAA*A	[58]
SplAP5.1	C*AAGCAGAAGACGGCATACGAGATAACGTGATGAG ATCGGTCTCGGCATTC*C	[58]
SplAP5.2	C*AAGCAGAAGACGGCATACGAGATAAACATCGGA GATCGGTCTCGGCATTC*C	[58]
SplAP5.3	C*AAGCAGAAGACGGCATACGAGATATGCCTAAGAG ATCGGTCTCGGCATTC*C	[58]
SplAP5.4	C*AAGCAGAAGACGGCATACGAGATAGTGGTCAGA GATCGGTCTCGGCATTC*C	[58]
SplAP5.5	C*AAGCAGAAGACGGCATACGAGATACCACTGTGAG ATCGGTCTCGGCATTC*C	[58]
SplAP5.6	C*AAGCAGAAGACGGCATACGAGATACATTGGCGA GATCGGTCTCGGCATTC*C	[58]
SplAP5.7	C*AAGCAGAAGACGGCATACGAGATCAGATCTGG AGATCGGTCTCGGCATTC*C	[58]
SplAP5.8	C*AAGCAGAAGACGGCATACGAGATCATCAAGTG AGATCGGTCTCGGCATTC*C	[58]
ForwardTnL	CTTAAGTTTGCTTCGATGACTGG	[58]
Transposon-specific primer	AATGATACGGCGACCACCGAGATCTACACCTGAATTACCCTGTTATCCCTATTTAGGTGAC	[59]
Sequencing primer	GACACTATAGAAGAGACCGGGGACTTATCAGC	[59]
